# Development and validation of headspace gas chromatography with a flame ionization detector method for the determination of ethanol in the vitreous humor

**DOI:** 10.1515/med-2024-1123

**Published:** 2025-01-15

**Authors:** Filip Mihajlović, Ivana Andrić, Živana Slović, Maja Vujović, Kristina Piskulić, Snežana Đorđević

**Affiliations:** Department of Pharmacology and Toxicology, Faculty of Medical Sciences, University of Kragujevac, Kragujevac, 34000, Serbia; Department of Forensic Medicine, Faculty of Medical Sciences, University of Kragujevac, Kragujevac, 34000, Serbia; Department of Forensic Medicine and Toxicology, University Clinical Center Kragujevac, Kragujevac, 34000, Serbia; Department of Pharmacy, Faculty of Medicine, University of Nis, Nis, 18000, Serbia; Institute of Forensic Medicine in Nis, Nis, 18000, Serbia; Department of Chemistry, Faculty of Science, University of Kragujevac, Radoja Domanovića 12, Kragujevac, 34000, Serbia; Department of Toxicological Chemistry, National Poison Control Center, Military Medical Academy, Belgrade, 11000, Serbia; Medical Faculty of the Military Medical Academy, University of Defense, Belgrade, 11000, Serbia

**Keywords:** alcohol, postmortem analysis, alternative samples, instrumental methods

## Abstract

**Introduction:**

Qualitative and quantitative testing of ethanol in *post-mortem* samples is an important analytical procedure that provides accurate, precise, and reliable results. Given the complexity of the issue, obtaining a realistic picture of lifelong alcoholemia requires supporting blood ethanol findings with analyses of alternative samples, primarily vitreous humor (VH).

**Objective:**

The objective of this study was to develop and validate a headspace gas chromatography with flame ionization detection (HS/GC-FID) method for determining ethanol concentration in VH.

**Materials and methods:**

Conditions for the HS/GC-FID method were established and the method was validated according to the guidelines of the European Medicines Agency. Validation parameters such as precision, accuracy, specificity, sensitivity, and linearity over a wide concentration range were evaluated through statistical analysis.

**Results:**

The method demonstrated precision, accuracy, specificity, and sensitivity. Additionally, it proved to be linear across a wide concentration range and relatively fast, making it suitable for rapid and routine determination of ethanol concentration in VH, particularly for forensic applications.

**Conclusion:**

Results from validation and application of the method to VH samples indicate that ethanol concentration in VH can be reliably determined using the presented HS/GC-FID method, making it a valuable tool in forensic investigations.

## 1 Introduction

Excessive consumption of alcoholic beverages and the state of intoxication always play a significant role in incidents, fatal injuries, drownings, suicides, and many other crimes registered by the police and emergency services [1]. Moreover, alcohol-induced intoxication is frequently the reason for accidents in the workplace, among family members, and in transport. There is also a strong link between alcohol consumption and violence.

Qualitative and quantitative testing of ethanol in postmortem samples is an analytical procedure that provides accurate, precise, and reliable results [2,3]. However, explaining postmortem results of blood alcohol concentration and drawing correct conclusions regarding antemortem levels, state of drunkenness, and degree of alcoholism at the time of death can be very complex [4,5,6]. The condition of the body, the time between death and autopsy, environmental conditions (temperature and humidity), and the nature of the samples collected for analysis are important factors to consider. Under some circumstances, alcohol may be produced after death due to microbial activity and glucose fermentation, which is a real problem if the corpse is in the stage of decomposition [7,8]. Postmortem diffusion of alcohol from the stomach is another aggravating factor if the person dies shortly after heavy drinking [9,10]. In addition, biological samples may be contaminated with ethanol or some other solvent during life-saving treatment [11]. Due to the complexity of the problem, to obtain a realistic picture of lifelong alcoholemia, the finding of alcohol in the blood should be supported by the analysis of alternative samples, primarily the vitreous humor (VH). Research shows that this analysis requires special attention from doctors and toxicologists when considering postmortem cases, especially when the process of decomposition of the body has begun, in the context of its importance for the legal consequences of a verified alcohol finding [12].

Bearing in mind the frequency of ethanol abuse, the increase in the number of acute and chronic ethanol poisonings, the importance of ethanol in forensic practice, and the state of the corpse during autopsies, determining the concentration of ethanol in the VH is of great forensic importance. Today, the method of choice for qualitative and quantitative analyses of ethanol in body fluids is gas chromatography (GC) with a flame ionization detector (FID), both in forensic toxicology and clinical laboratories, using headspace (HS) sampling [13,14]. The aim of this article was the development and introduction of an analytical method of GC with an FID detector (GC/FID) for the routine determination of ethanol in the VH samples.

## 2 Materials and methods

### 2.1 Basic principles

We used the internal standard method for the quantification of ethanol in VH. For the internal standard, we chose *n*-propanol, which, with ethanol, has, in a wider temperature interval, a constant vapor pressure. Ethanol from VH is converted into a gas phase by heating in a hermetically sealed bottle. A part of the gaseous phase is introduced into the gas chromatograph, and after separation on a column of the appropriate polarity, it is detected by an FID.

### 2.2 Chromatographic system and conditions

The chromatographic separation and quantification of the ethanol were performed on a Hewlett Packard 5890 series II Gas Chromatograph with an HS sample injection device: (Hewlett Packard HS sampler 19395A) and flame-ionizing detector heated to 2,600°C. The temperature in the autosampler is set to 850°C. We used Zebra BAC1, 30 m × 0.53 mm ID column for separation. The carrier gas is nitrogen, where the nitrogen flow rate is 30 mL/min, hydrogen 40 mL/min, and air 400 mL/min.

### 2.3 Chemicals and reagents

For the preparation of calibration solutions and control samples, as well as standard ethanol solutions for selectivity testing, we used 96% ethanol with a density of 0.801 kg/m^3^. In addition to ethanol, for research purposes, we used *n*-propranol and distilled water of liquid chromatography with mass spectrophotometry grade. All reagents were obtained from the same manufacturer (Merck, Darmstadt, Germany). The basic standard ethanol solution with a concentration of 10 mg/mL was obtained by dissolving 1.18 mL of 96% ethanol in a 100 mL measuring vessel. In the further procedure, standard solutions of ethanol in water were made in the following concentrations: 0.2, 0.5, 0.75, 1.0, and 2.5 mg/mL.

### 2.4 Preparation of VH samples

Pool samples for the preparation of loaded VHs, which were used for method validation, were obtained from seven deceased patients in whom no ethanol was detected at the time of death. Vitreous humor from cadaver material was obtained by puncturing the wall of the eyeball in the area of the outer corner of the eyelid with a sterile, thin needle. The samples were taken during the forensic autopsy at the Department of Forensic Medicine and Toxicology, University Clinical Center Kragujevac. The VH samples were placed in test tubes, adequately transported, then transferred to the toxicological laboratory, and homogenized in a beaker. Until analysis, the samples were stored at −20°C.

### 2.5 Preparation of vitreous samples loaded with ethanol and determination procedure

Ethanol-loaded VH samples were obtained by measuring a certain volume of ethanol working solution 10.0 mg/mL and VH, to obtain loaded VH solutions in concentrations: 0.2, 0.5, 0.75, 1.0, and 2.5 mg/mL. The further process of determining and preparing the samples involved measuring 200 µL of loaded VH solution samples and 2,000 µL of the internal standard n-propanol into a 10 mL glass vial. The samples are then closed with a rubber and metal closure, hermetically, with the help of forceps, and such samples are ready for further analysis. The samples are heated before injection, and this incubation period enables the establishment of a dynamic balance of ethanol concentrations in the blood and vapor above the liquid sample. Then, automatic injection of the sample is performed using the HS technique, and in this way, the gas phase is introduced into the GC column.

### 2.6 Method validation

Method validation was carried out in accordance with the official European Medicines Agency (EMA) [15] guideline for bioanalytical method validation through the assessment of the following validation parameters: selectivity, linearity of the method and working range, limit of detection (LOD), limit of quantification (LOQ), precision, accuracy, and stability.

#### 2.6.1 Selectivity

Selectivity represents the ability of the method to determine exactly the specific investigated substance (analyte of interest) without interference from other components that can be found in the analyzed sample. This parameter shows and confirms the possibility of separation, identification, and quantification of the analyte of interest in relation to other analytes present in the sample (if any). The selectivity was tested by comparing the chromatograms of five blank ethanol samples and five loaded samples of VH, in concentrations that are of the greatest forensic importance (0.20, 0.50, 0.75, 1.0, and 2.50 mg/mL).

#### 2.6.2 Linearity

To test linearity in the selected range of concentrations (from 0.001 to 2.50 mg/mL) and obtain calibration standards, a series of standard ethanol solutions were measured and analyzed by GC, and the response of the detector was registered over the area of the peak characteristic of the analyzed compound. Based on the obtained results, the parameters of the regression equation (slope (*a*), intercept, and correlation coefficient) were determined. For each concentration, three consecutive measurements were performed.

#### 2.6.3 LOD

The LOD is the lowest concentration of the tested substance that can be detected by an analytical method but not quantified with particular precision and accuracy. It is the lowest concentration that differs from the baseline signal. The LOD was determined by calculating the standard deviation of the lowest point of the calibration curve (0.15 mg/mL) during the linearity test according to the following formula: LOD = (3.3 × standard deviation [SD])/*a*.

#### 2.6.4 LOQ

The LOQ is the lowest concentration that can be reliably determined by an analytical method. The LOQ was determined by calculating the standard deviation of the lowest point of the calibration curve (0.15 mg/mL) during the linearity test according to the following formula: LOD = (10 × SD)/*a*.

#### 2.6.5 Precision

The repeatability of the method is one way of expressing its precision under the same working conditions in a short time interval. It shows the intra-laboratory variability obtained in a short time interval, with one analyst on the same equipment. Repeatability represents the closeness of agreement between mutually independent test results and is expressed in the form of a standard deviation or relative standard deviation. The relative standard deviation or coefficient of variation, as it is otherwise called, can be different in cases where the influence of concentration should be eliminated because it is independent of it. The reproducibility of the method is determined by preparing ten standard samples of ethanol with a concentration of 1.0 mg/mL and by chromatography.

#### 2.6.6 Accuracy

The recovery test was used to test the accuracy of the method. Three samples of standard solutions of ethanol in VH were measured for five concentration levels in the range of the calibration curve for ethanol. The measured concentrations were compared with the expected ones. The ratio between the obtained and expected concentrations was calculated and expressed as a percentage, which represents the recovery value.

#### 2.6.7 Stability

As part of the validation of the method, the short-term stability of the samples and working solutions, the stability of the autoinjector, and the stability of the sample during freezing and thawing were examined. To test the short-term stability of the samples, the stability in the autoinjector, as well as the stability after the freezing and thawing processes, a sample “loaded” with ethanol at a concentration of 5 mg/mL was used. All samples were made in triplicate. Short-term stability was evaluated by comparing freshly prepared samples with samples that had been standing at room temperature for 4 h. Stability during freezing and thawing was evaluated after three cycles, with the period between two cycles being from 12 to 24 h. Stability in the autoinjector was assessed by analyzing samples at the beginning and end of the analytical sequence.

The stability of the working solutions, stored in a refrigerator at a temperature of 2–8°C, was tested for a period of 1 month. The obtained results were compared, whereby the allowed deviation for the stability of the samples and the working solution is 15%, in accordance with the recommendations of the guide for the validation of bioanalytical methods of the EMA [15].

#### 2.6.8 Measurement uncertainty

In accordance with the procedure of assessment and determination of measurement uncertainty, all its potential sources were considered. Quantification of sources of uncertainty was done so that the individual contributions of each source were expressed as standard deviations, and they are:

1. **The concentration of the ethanol reference standard (** ***u*** **1)** – according to the certificate provided by the manufacturer, the concentration of the ethanol reference standard is 1.002 ± 0.034. The measurement uncertainty of the measurement results is expressed as an expanded measurement uncertainty, which is obtained by multiplying the combined measurement uncertainty by the factor *k* = 2, which for a normal distribution corresponds to a confidence interval of 95% and is 0.034;
2. **Measurement uncertainty of the pipette (** ***u*** **2)** – according to the ethanolization certificate provided by the accreditation laboratory for ethanolization, the measurement uncertainty of the measurement results is expressed as an expanded measurement uncertainty, which is obtained by multiplying the combined measurement uncertainty by the factor *k* = 2, which for a normal distribution corresponds to a confidence interval of 95% and for a 1,000 µL pipette is 0.0064, and for a 200 µL pipette is 0.0009;
3. **Measurement uncertainty of the instrument (** ***u*** **3)** – the measurement uncertainty of the instrument, which is confirmed by the authorized service, is given for the reproducibility of surfaces (relative standard deviation [RSD] = 1.72%) and retention time (RSD = 0.012%);
4. **Ethanol concentration from the calibration curve (** ***u*** **4)** – the uncertainty of the concentration read from the calibration curve is defined with the standard deviation from several repeated readings of the same sample. Data for method validation were used for this measurement uncertainty parameter. An ethanol solution with a concentration of 1.0 mg/mL was passed through the complete measurement procedure ten times. The calculated mean value of ethanol was 1.03203 mg/mL, and the obtained real standard deviation was 0.5672%.
5. **Recovery (** ***u*** **5)** – determining the measurement uncertainty that comes from recovery includes the uncertainty of the measurement conditions, the influence of temperature, humidity, pressure, the uncertainty of extracting the analyte from the matrix, and the uncertainty of repeatability. The measurement uncertainty arising from the recovery (yield) was calculated using data for method validation, as the standard deviation of the recovery values obtained in the repeated preparation procedure.

After quantifying all sources of uncertainty and expressing them as standard uncertainty, the combined standard measurement uncertainty (*u*) for the method of determining ethanol in the vitreous fluid as the root of the sum of the squares of the individual components was calculated. The combined measurement uncertainty for the method of determining ethanol in the vitreous fluid is *u* = 1.80%. The results of the determination of ethanol will be expressed with the total measurement uncertainty of the method (*U*), which is obtained as a result of multiplying the combined measurement uncertainty (*u*) and a numerical factor (*k*) that is 2. The reason for calculating the expanded measurement uncertainty is to achieve a sufficiently high confidence (on average 95%) that the true value lies within the interval determined by the measurement result. For this method, the total measurement uncertainty is *U* = 3.60%.


**Ethical approval:** The samples were taken after receiving the decision of the Ethics Committee of the University Clinical Center Kragujevac (No. 01/22-433).

## 3 Results

**Figure 1:**
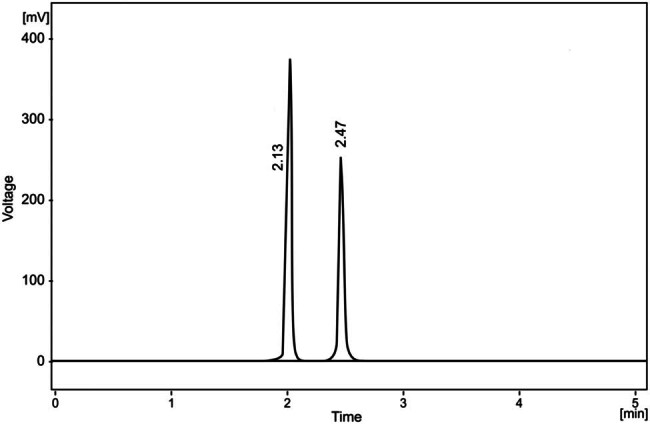
Representative chromatogram of ethanol in VH.

**Figure 2:**
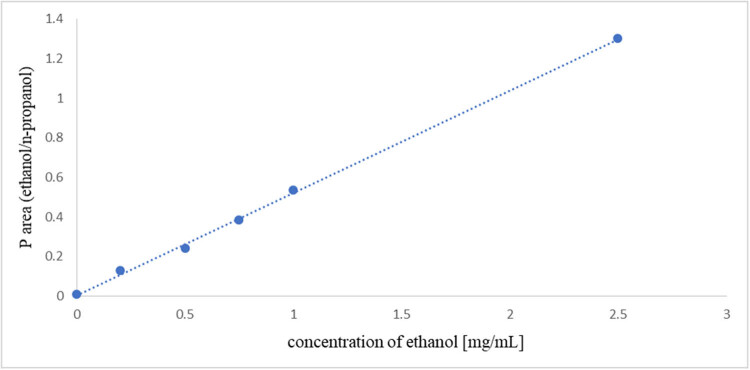
Calibration curve.

The results showed that under our chromatography conditions, the average retention time for ethanol is 2.13 min, while for *n*-propanol it is 2.47 min. As shown in Figure 1, a representative chromatogram of VH obtained by the HS/GC-FID method is presented. The equation of the calibration curve is *y* = 0.5174*x* + 0.0039, the correlation coefficient is *r* = 0.9989. The calibration curve is presented in Figure 2. The linearity of an analytical method is defined as the direct proportionality between the measurement results (detector response, i.e., the area under the curve) and the analyte concentration in the sample, within a defined concentration range. Linearity is evaluated by calculating the regression curve using the least squares method and is expressed through the correlation coefficient (*r*), *y*-intercept, and slope of the regression curve. The equation for the calibration curve takes the form: *y* = *ax* + *b*, where:

- *x* represents the concentration of the analyte (ethanol),
- *y* corresponds to the ratio of the area under the curve of the analyte to that of the internal standard (*n*-propanol),
- *a* is the intercept on the *y*-axis (the value of *y* when *x* = 0), and
- *b* is the regression coefficient.

The working measurement range is 0.15–2.50 mg/mL. Figure 1 shows the graph of the calibration curve of ethanol where the peak area ratio of ethanol/*n*-propanol was plotted versus the concentration of the respective six calibration standards. Table 1 shows concentrations of standard ethanol solutions, obtained surfaces, and parameters of regression equations. Table 1 presents the investigation of linearity within the selected concentration range (0.2–2.5 mg/mL) to obtain calibration curves. A series of standard ethanol solutions were prepared and analyzed using GC. The detector response was recorded based on the peak area characteristic of the analyzed compound. From the obtained results, the parameters of the regression equation, including the slope, intercept, and correlation coefficient, were determined. For each concentration, three consecutive measurements were conducted.

**Table 1:** Concentrations of standard ethanol solutions, obtained surfaces, and parameters of regression equations

Concentration of standard solution of ethanol (mg/mL)	*P* area of ethanol	Average value of *P* area of ethanol	*P* area of *n*-propanol (internal standard)	Average value of *P* area of *n*-propanol (internal standard)	Average value of *P* area ethanol/*n*-propanol
0.20	187,942	191,615	767,480	829,475	0.22
	188,569		855,416		
	198,334		865,531		
0.50	752,278	744,180	819,598	813,675	0.91
	745,715		812,677		
	734,549		808,751		
0.75	1,195,872	1,217,427	805,509	813,994	1.49
	1,201,679		800,410		
	1,254,730		836,064		
1.00	1,506,794	1,480,198	796,929	786,055	1.88
	1,436,882		768,451		
	1,496,920		792,785		
2.50	3,021,305	2,984,733	793,352	779,456	3.82
	3,011,825		790,450		
	2,921,070		754,568		

The calculated LOD was 0.005 mg/mL, while the LOQ was determined to be 0.017 mg/mL, indicating that the method is highly accurate. The method’s accuracy is presented in Table 2, which also highlights method repeatability, a key indicator of precision under consistent operating conditions over a short period. This reflects intra-laboratory variability within a brief timeframe, with one analyst using the same equipment. Repeatability, which represents the degree of agreement between independent test results, is quantified using standard deviation or relative standard deviation. The relative standard deviation, also known as the coefficient of variation, can vary in instances where the influence of concentration needs to be minimized, as it is independent of concentration. Method repeatability was assessed by preparing ten ethanol standard samples at a concentration of 1.0 mg/mL and performing chromatographic analysis. The mean recovery values (mean ± SD) for all five tested concentrations are provided in Table 3. This table details the results of the recovery test conducted to evaluate method accuracy. Three samples of ethanol standard solutions in VH were analyzed at five different concentration levels along the ethanol calibration curve. Measured concentrations were compared to the expected values, and the ratio between the two was calculated and expressed as a percentage, representing the recovery value. To complete the analysis, additionally, the mean values, standard deviations, and relative standard deviations were determined. All validation parameters are summarized in Table 4.

**Table 2:** Examination of the accuracy of the method for the determination of ethanol in the VH

Expected concentration (mg/mL)	Determined concentration (mg/mL)	Recovery (%)
1.00	1.0437	104.37
1.00	1.0285	102.85
1.00	1.0285	102.68
1.00	1.0368	103.11
1.00	1.0311	103.28
1.00	1.0288	102.88
1.00	1.0276	102.76
1.00	1.0428	104.28
1.00	1.0281	102.81
1.00	1.0301	103.01
SD = 0.005854		
RSD (%) = 0.5672		

SD – standard deviation; RSD – relative standard deviation.

**Table 3:** Recovery test

	Number of measurement	Real concentration (mg/mL)	Measured concentration (mg/mL)	Mean value	Accuracy (%)
	I	0.20	0.201	0.201	101.50
	II	0.20	0.202		101.00
	III	0.20	0.200		100.00
	I	0.50	0.502	0.499	100.46
	II	0.50	0.493		98.62
	III	0.50	0.502		100.38
	I	0.75	0.751	0.750	100.13
	II	0.75	0.750		100.00
	III	0.75	0.749		99.87
	I	1.00	0.996	0.992	99.56
	II	1.00	1.002		100.20
	III	1.00	0.978		97.79
	I	2.50	2.510	2.511	100.40
	II	2.50	2.511		100.44
	III	2.50	2.512		100.48
**Accuracy of method**					
	Accuracy for concentration of 0.20 mg/mL	Accuracy for concentration of 0.50 mg/mL	Accuracy for concentration of 0.75 mg/mL	Accuracy for concentration of 1.00 mg/mL	Accuracy for concentration of 2.50 mg/mL
	100.50	100.46	100.13	99.56	100.44
	100.00	98.62	100.00	100.20	100.48
	101.00	100.38	99.87	97.79	100.40
Mean value	100.50	99.82	100.00	99.18	100.44
Accuracy (80–120%)	100.11				
SD	0.656				
%RSD	0.655				
95% limit	0.504				

SD – standard deviation; RSD – relative standard deviation.

**Table 4:** Presentation of the obtained parameters of the validation study for the determination of ethanol in the vitreous fluid

Results of validation tests	
Operating range	0.15–2.5 mg/mL
The correlation coefficient	*r* = 0.9989
Calibration curve equation	*y* = 0.5174*x* + 0.0039
Detection limit	0.005
LOQ	0.017
Precision	0.00572
Accuracy	100.11
Stability	15%
Measurement uncertainty	3.6%

## 4 Discussion

The analytical methods to detect the volatile substance in *post-mortem* samples, such as peripheral or central blood, VH, kidney, brain, liver, and urine, have long been studied in forensic literature [16,17]. Ethanol analysis by HS/GC-FID is well established in the literature [18]. In combination with appropriate detectors, it enables the identification and quantification of a wide range of toxic agents from gases, organic converters, and pesticides to a wide variety of natural and synthetic drugs and medicines [19].

When HS/GC-FID is used for quantitative analysis, it is necessary to minimize or eliminate the matrix effect [20], which was achieved by adding small amounts of NaCl. The best way to eliminate the matrix effect is to saturate the biological sample with an inorganic salt such as NaCl [21]. This salting-out technique allows for an increase in the vapor pressure of the non-electrolyte (ethanol) in the vial and increases the sensitivity of the HS/GC-FID analysis. The method of salting out is not recommended in everyday practice, but it can be used if extremely small amounts of volatile substances in the sample are analyzed and it does not affect the result. Biological samples for ethanol content should be analyzed in duplicate on two different chromatographic systems that would provide different retention times for ethanol and internal standards. Some laboratories recommend using two different internal standards, e.g., *n*-propanol and *t*-butanol. A small amount of n-propanol can be produced during the process of decay and putrefaction [22], but in this work, VH samples obtained from rotting corpses were not used for the analysis of ethanol concentration. However, recent research [23] has shown that *n*-propanol is not produced in VH after death, which supports the use of *n*-propanol as an internal standard for the validation of this method.

Using highly specific GC for analysis, ethanol can be reliably determined in the presence of potentially interfering substances (e.g., acetaldehyde, ethyl acetate, 2-propanol, *n*-butanol) that may be produced during body decomposition. Low values of blood alcohol concentrations in *post-mortem* samples (<30 mg/100 mL or 0.30 mg/mL) are debatable without supporting evidence of ethanol analysis in vitreous fluid and/or urine [24]. High values of ethanol concentration in VH (<2.00 mg/mL) can be a consequence of various circumstances of death (fires, combined causes of poisoning), so high ethanol values in VH are supported by the analysis of ethanol in other body fluids.

The advantage of this work is that the presented method is validated by all validation parameters, overlooked by the *International Organization for Standardization/International Electrotechnical Commission* (ICO/IEC 17025) [25]. Compared to other studies [26,27], the results of this article match those of others, when it comes to the dynamic range, regression coefficient, LOD, and LOQ. In the study by Chun et al. [27], it was shown that the results regarding the stability of the method were identical to the results obtained for the same standard and conditions. The literature search so far did not find any publications on this topic, which examined the measurement uncertainty of the method as part of the validation. In addition to ethanol, the method is suitable for the quantification of methanol and formaldehyde in VH [28]. It may be useful for future research, according to the diversity of the circumstances of death in forensic medicine. The validated method offers a distinct advantage by incorporating the calculation of measurement uncertainty, which traditional methods do not have as data. Considering that VH is the most common alternative sample in forensic toxicology, the precision of the chosen analytical method is of great importance in that case. Therefore, it is important to determine all types of measurement uncertainty of the method, which include measuring instruments, laboratory vessels, and accessories and apparatus. Through the determination of both total and combined measurement uncertainty, the influence of analytical and post-analytical factors contributing to potential errors has been minimized. Notably, previous studies on this subject [28,29] have not addressed the issue of measurement uncertainty. How much more precise the set method is compared to traditional methods that have been validated for the analysis of ethanol in VH is best shown by the accuracy of 0.005721.

## 5 Conclusion

With this work, we have developed a reliable, precise, and accurate HS/GC-FID method for the identification and quantitative analysis of ethanol in the VH. The introduced method represents an important contribution to the work of the forensic toxicology laboratory and provides significant help in obtaining a realistic picture of life-long alcoholism, when, according to the order of the prosecution, the finding of ethanol in the blood needs to be supported by the analysis of ethanol in one of the alternative samples, first of all, the VH.
